# Perovskite Oxynitride Solid Solutions of LaTaON_2_‐CaTaO_2_N with Greatly Enhanced Photogenerated Charge Separation for Solar‐Driven Overall Water Splitting

**DOI:** 10.1002/advs.202003343

**Published:** 2020-11-25

**Authors:** Yawei Wang, Yuyang Kang, Huaze Zhu, Gang Liu, John T. S. Irvine, Xiaoxiang Xu

**Affiliations:** ^1^ Clinical and Central Lab Putuo People's Hospital Shanghai Key Lab of Chemical Assessment and Sustainability School of Chemical Science and Engineering Tongji University Shanghai China; ^2^ Shenyang National Laboratory for Materials Science Institute of Metal Research Chinese Academy of Sciences 72 Wenhua Road Shenyang 110016 China; ^3^ School of Materials Science and Engineering University of Science and Technology of China 72 Wenhua Road Shenyang 110016 China; ^4^ School of Chemistry University of St Andrews St Andrews Fife KY16 9ST UK

**Keywords:** oxynitrides, perovskites, solid solution, visible light, water splitting

## Abstract

The search for solar‐driven photocatalysts for overall water splitting has been actively pursued. Although metal oxynitrides with metal d^0^/d^10^‐closed shell configuration are very promising candidates in terms of their visible light absorption, they usually suffer from serious photo‐generated charge recombination and thus, little photoactivity. Here, by forming their solid solutions of LaTaON_2_ and CaTaO_2_N, which are traditionally considered to be inorganic yellow‐red pigments but have poor photocatalytic activity, a class of promising solar‐driven photocatalysts La_1‐_ *_x_*Ca*_x_*TaO_1+y_N_2‐_ *_y_* (0 ≤ *x*, *y* ≤ 1) are explored. In particular, the optimal photocatalyst with *x* = 0.9 has the ability of realizing overall water splitting with stoichiometric H_2_/O_2_ ratio under the illumination of both AM1.5 simulated solar light and visible light. The modulated key parameters including band structure, Ta bonding environment, defects concentration, and band edge alignments revealed in La_0.1_Ca_0.9_TaO_1+_ *_y_*N_2‐_ *_y_* have substantially promoted the separation of photogenerated charge carriers with sufficient energetics for water oxidation and reduction reactions. The results obtained in this study provide an important candidate for designing efficient solar‐driven photocatalysts for overall water splitting.

## Graphical abstract

Forming solid solutions between LaTaON_2_ and CaTaO_2_N leads to a series of promising solar‐driven photocatalysts La_1‐_ *_x_*Ca*_x_*TaO_1+_ *_y_*N_2‐_ *_y_* (0 ≤ *x*, *y* ≤ 1). Overall water splitting with stoichiometric H_2_/O_2_ ratio can be achieved at optimal composition of *x* = 0.9 under the illumination of both AM1.5 simulated solar light and visible light.

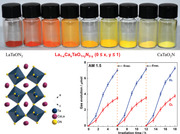

Water splitting into H_2_ and O_2_ on particulate photocatalysts stands as one of the simplest and most cost‐effective techniques for storing solar energy into chemical fuels.^[^ ^1^ ^]^ With easy access to both reactant (water) and energy input (solar insolation), this technique signifies a promising means to setup a clean and sustainable energy infrastructure that can eventually decarbonize our fossil‐fuel based energy economy.^[^ ^2^ ^]^ For instance, large water splitting panels fabricated from particulate photocatalyst has shown to be a viable means for large‐scale production of low‐cost renewable solar hydrogen.^[^ ^3^ ^]^ Although efficient overall water splitting has been realized on wide bandgap semiconductors using ultra‐violet (UV) photons,^[^ ^4^, ^5^ ^]^ visible‐light‐driven overall water splitting has been rather difficult, occurring to be the bottleneck problem for efficient solar energy conversion.^[^ ^6^, ^7^ ^]^ Thereby, exploring photocatalysts sensitive to visible light photons for overall water splitting becomes a premier target.^[^ ^8^ ^]^

Previous reports on single‐absorber type photocatalyst for overall water splitting using visible light photons are almost exclusively referred to metal oxynitrides with *d* ^10^ or *d*° closed‐shell electronic structure, such as (Ga_1‐_ *_x_*Zn*_x_*)(N_1‐_ *_x_*O*_x_*),^[^ ^9^ ^]^ (Zn_1+_ *_x_*Ge)(N_2_O*_x_*),^[^ ^10^ ^]^ TaON,^[^ ^11^ ^]^ Ta_3_N_5_,^[^ ^12^ ^]^ LaMg*_x_*Ta_1‐_ *_x_*O_1+3_ *_x_*N_2‐3_ *_x_* ^[^ ^13^ ^]^ etc. Fine composition/structure tuning as well as peculiar surface modifications is often indispensable to realize stoichiometric H_2_ and O_2_ evolution.^[^ ^10^, ^14^ ^]^ This can be rationalized by a need to minimize energy barriers for charge migration and transfer so as to compensate the reduced energetics of charges generated by visible light photons.^[^ ^15^ ^]^ This imposes stringent standard on the type of crystal structures upon which various modifications can be performed. Perovskite oxynitrides are emerging groups of functional materials that show potential applications in a wealth of fields including colossal magnetoresistivity, dielectrics, pigments, phosphors, etc.^[^ ^16^ ^]^ Their perovskite type structures are extremely flexible and tolerant to diverse cation/anion displacements,^[^ ^17^, ^18^ ^]^ opening ample playgrounds to tailor the desired physicochemical properties.

Perovskite oxynitrides with chemical formula AM(O,N)_3_ (A = La, Ca, Sr, Ba; M = Ti, Nb, Ta) have bandgap ranging from 1.7 to 2.6 eV, showing strong absorption in the visible light region.^[^ ^19^ ^]^ However, their photocatalytic activity for water splitting is normally very poor even with an aid of sacrificial agents.^[^ ^20^, ^21^, ^22^, ^23^, ^24^ ^]^ This has been generally ascribed to several key factors that are strongly linked to charge separation, migration, and transfer:^[^ ^12^, ^20^, ^25^, ^26^ ^]^ i) Defects generated during synthetic procedures (high temperature ammonolysis) as their presence induces rapid charge recombination events; ii) structural distortions (tilting of M(O,N)_6_ octahedrons) which prevent fast charge migrations; iii) improper band edge alignments which result in incommensurate energetics for water reduction and oxidation half‐reactions. How to properly tackle these problems is a prerequisite to achieve visible‐light water splitting on these compounds and still remains a great challenge.

In this work, solid solutions between LaTaON_2_ and CaTaO_2_N were managed to finely tune their optical absorption, Ta bonding environment, defects concentration, and band edge alignments. The successful manipulation of these important parameters offers us flexible modifications on their photocatalytic activities. At optimal conditions, visible light driven overall water splitting has been achieved on this material system for the first time, to our best knowledge.

Typical synthesized sample powders with a variation of Ca content are shown in Figure S1a, Supporting Information, detailed synthetic and analytic information can be found in the Supporting Information. The formation of solid solutions La_1‐_ *_x_*Ca*_x_*TaO_1+_ *_y_*N_2‐_ *_y_* (0 ≤ *x*, *y* ≤ 1) is confirmed by their X‐ray powder diffraction (XRD) patterns that closely resemble standard ones of LaTaON_2_ and CaTaO_2_N. All reflections consecutively shift toward high angles along with Ca uptake (Figure S1a, Supporting Information), indicating continuous shrinkage of LaTaON_2_ unit cell by dissolving CaTaO_2_N. This is further verified by Rietveld refinements of their XRD data (**Figure** **1**a, Table S1, Supporting Information) and is in a good agreement with vegard's law as La^3+^/N^3−^ ions are gradually replaced by smaller Ca^2+^/O^2−^ ions in the structure.^[^ ^27^ ^]^ La/Ca probably has random distributions at the A sites of perovskite as super‐lattice reflections from cation ordering are not observed. Nevertheless, there is a watershed point around *x* = 0.7 whereby space group changes from *I12/m* to *Pnma* (Figure S1b, Supporting Information). Such a symmetry change from monoclinic to orthorhombic likely stems from the tilt of Ta(O, N)_6_ octahedrons in a manner that is gradually increased from LaTaON_2_ (Glazer notion: *a^−^b^−^c^0^*) to CaTaO_2_N (Glazer notion: *a^−^b^−^c^+^*).^[^ ^17^, ^22^ ^]^ This can be seen from the refined crystal structures of LaTaON_2_, La_0.1_Ca_0.9_TaO_1+_ *_y_*N_2‐_ *_y_* (*x* = 0.9) and CaTaO_2_N (Figure 1a). For these Ta‐based perovskite oxynitrides, smaller unit cell generally means shorter Ta—O/N bonds whilst increased tilt of Ta(O, N)_6_ octahedrons often indicates decreased Ta—O/N—Ta bond angles that is more deviated from 180° (see Table S1, Supporting Information). These two factors are of critical importance to their electronic structures since the hybridizations between Ta 5*d* and O/N 2*p* orbitals contribute mostly to the conduction and valence bands.^[^ ^26^, ^28^, ^29^ ^]^ For instance, shorter Ta—O/N bonds favor *p‐d σ* bonds while deviation of Ta—O/N—Ta bond angles away from 180° increases the difficulties for *p‐d π* bonds formation.^[^ ^28^, ^30^ ^]^ Thereby, forming solid solutions between LaTaON_2_ and CaTaO_2_N is a useful tool to finely tune their electronic structures which govern the behavior of photo‐generated charges under light illuminations.

**Figure 1:**
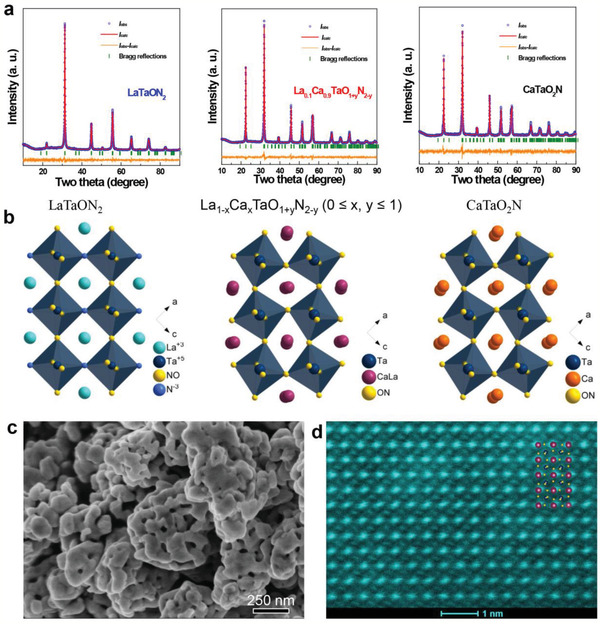
a) Observed and calculated X‐ray powder diffraction patterns of LaTaON_2_, La_0.1_Ca_0.9_TaO_1+_ *_y_*N_2‐_ *_y_* (*x* = 0.9) and CaTaO_2_N; the refinements all converge with reasonable *R* factors and *χ^2^* factor (LaTaON_2_: *R*_p_ = 3.76%, *R*_wp_ = 4.91%, *χ^2^* = 1.311; La_0.1_Ca_0.9_TaO_1+_ *_y_*N_2‐_ *_y_* (*x* = 0.9): *R*_p_ = 4.94%, *R*_wp_ = 6.37%, *χ^2^* = 2.762; CaTaO_2_N: *R*_p_ = 5.58%, *R*_wp_ = 6.91%, *χ^2^* = 2.835). b) Schematic illustration of crystal structures of LaTaON_2_ (left panel), La_0.1_Ca_0.9_TaO_1+_ *_y_*N_2‐_ *_y_* (*x* = 0.9) (middle panel) and CaTaO_2_N (right panel), these structures are projected along [010] direction. c) Field emission scanning electron microscopy images of La_0.1_Ca_0.9_TaO_1+_ *_y_*N_2‐_ *_y_* (*x* = 0.9). d) High‐angle annular dark‐field scanning transmission electron microscopy (HAADF‐STEM) image of La_0.1_Ca_0.9_TaO_1+_ *_y_*N_2‐_ *_y_* (*x* = 0.9) along [010] direction. Inset: Depicted crystal structure of La_0.1_Ca_0.9_TaO_1+_ *_y_*N_2‐_ *_y_* (*x* = 0.9) projected along [010] direction.

All sample powders are composed of particles with particle size around 500 nm (Figure S2a–c, Supporting Information). These particles contain interconnected granules of 40–100 nm in size according to their particle size distribution (Figure S3a, Supporting Information) and own highly porous microstructures according to their SEM and TEM images (Figure 1c, Figure S2, Supporting Information). Such porosity is responsible for their large surface area (≈13 m^2^·g^−1^) and originates the commonly observed structural shrinkage occurred in the transition from metal oxides to metal oxynitrides or metal nitrides during the high temperature ammonolysis.^[^ ^31^ ^]^ Porous microstructures have been very useful to improve photocatalytic activity of oxynitrides and are generally introduced by altering preparation methods.^[^ ^32^, ^33^ ^]^ The pore size of all samples mostly falls into mesoporous range (2–50 nm) according to BET analysis (Figure S3b, Supporting Information), which is beneficial for mass transportations in water splitting reactions. Further analysis under HAADF‐STEM conditions suggests the high crystallinity of the oxynitride particles. A typical HAADF‐STEM image of La_0.1_Ca_0.9_TaO_1+_ *_y_*N_2‐_ *_y_* (*x* = 0.9) in Figure 1d illustrates the random occupation of La/Ca at A sites which matches well with crystal structures from Rietveld refinement (see inset in Figure 1d).

Although sharing similar crystal structures and microstructures, these sample powders have distinct colors that are strongly linked to the content of Ca at A sites. The color of sample powders varies sequentially from red, orange, yellow to light yellow as the Ca content increases from 0 (LaTaON_2_) to 100 at% (CaTaO_2_N), being potential applications as non‐toxic pigments.^[^ ^34^ ^]^ This is further verified by their UV–vis diffuse reflectance spectroscopy where the absorption edge of solid solutions has a continuous blue‐shift from 650 nm for LaTaON_2_ to 500 nm for CaTaO_2_N (**Figure** **2**a). Correspondingly, the direct band gap of solid solutions can be finely tuned between 1.95 and 2.47 eV simply by controlling the Ca content at A sites according to Tauc plot (Figures S4 and S5, Table S2, Supporting Information). This phenomenon most probably arises from a change on O/N ratio and gradual increase on the level of structure distortions.^[^ ^26^, ^34^ ^]^ This peculiar property is quite useful as it offers us freedoms in manipulating the energetics of photo‐generated charges if compared with conventional semiconductors whose band gap is fixed. The band edge positions of solid solutions La_1‐_ *_x_*Ca*_x_*TaO_1+_ *_y_*N_2‐_ *_y_* (0 ≤ *x*, *y* ≤ 1) were revealed by a combination of Mott–Schottky (MS) analysis in conjunction with X‐ray photoelectron spectroscopy (XPS) valence band scan. Their MS plots illustrated in Figure S6, Supporting Information, suggest the flat band potential changes from −0.49 to −0.92 V versus NHE along with Ca content. XPS valence band scan informs that the energy gap between valence band edge and Fermi level for these samples varies from 1.45 to 2.02 eV (Figure 2b). Considering their band gap, a picture of their band‐edge positions is schematically illustrated in Figure 2c. It can be seen from the figure that continuous band edge alignment can be realized simply by adjusting the composition of solid solutions. Compared with LaTaON_2_ and CaTaO_2_N, the band edge positions of La_0.1_Ca_0.9_TaO_1+_ *_y_*N_2‐_ *_y_* (*x* = 0.9) have been adjusted in a manner that a high driving force of ≥ 0.5 V is available for both water reduction and oxidation reactions. This is of critical importance for overall water splitting on photocatalysts of single‐light semiconducting absorber which has both water reduction and oxidation reactions occurring on single particle.

**Figure 2:**
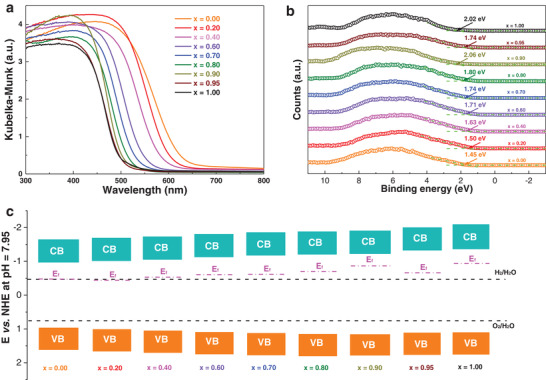
a) Ultraviolet‐visible diffuse reflectance spectroscopy (UV–vis DRS) of La_1‐_ *_x_*Ca*_x_*TaO_1+_ *_y_*N_2‐_ *_y_* (0 ≤ *x*, *y* ≤ 1). b) XPS valence band scan of La_1‐_ *_x_*Ca*_x_*TaO_1+_ *_y_*N_2‐_ *_y_* (0 ≤ *x*, *y* ≤ 1). c) Schematic illustration of the band structures of La_1‐_ *_x_*Ca*_x_*TaO_1+_ *_y_*N_2‐_ *_y_* (0 ≤ *x*, *y* ≤ 1) referring to water redox potentials.

In addition, Raman spectra (Figure S7, Supporting Information) reveal interesting features on local bonding environment in solid solutions. Raman peak assigned to the breathing mode of Ta(O, N)_6_ octahedrons shifts from 795 to 828 cm^−1^ as Ca content increases in solid solutions.^[^ ^35^ ^]^ This implies the enhanced Ta—O/N bond strength or increased crystal field in Ta(O, N)_6_ octahedrons along with Ca uptake. A stronger Ta—O/N bond or crystal field in Ta(O, N)_6_ octahedrons helps stabilize Ta at its high oxidation state which in turn inhibits defects such as Ta^4+^ species.^[^ ^36^ ^]^ This deduction is confirmed by XPS analysis whereby Ta^4+^ 4*f* signals centered at around 24.2 and 26.3 eV^[^ ^37^ ^]^ decrease clearly as Ca content increases in solid solutions (Figure S9, Supporting Information). Ta^4+^ species is generally detrimental to photocatalytic activity as they may trap photo‐generated charges and accelerate charge recombination.^[^ ^26^, ^38^ ^]^ As anticipated, the Ca uptake is accompanied by the decrement of N content in solid solutions, which is affirmed by XPS (Figure S9, Supporting Information) as well as thermogravimetric analysis (Figure S8, Table S2, Supporting Information). All other constituent elements such as O, La, and Ca have reasonable XPS bonding states (Figure S9, Supporting Information) and are consistent with literatures.^[^ ^5^, ^39^, ^40^ ^]^

As the key parameters including the bandgap, Ta—O/N bonding environment and defects content vary much among solid solutions, their photocatalytic activity was evaluated first by investigating two half‐reactions of water splitting before optimal conditions were screened out for subsequent overall water splitting reactions. Control experiments that lack one of the following components such as sample powders, water, and light illumination did not produce detectable H_2_/O_2_, thereby ruling out possible spontaneous water reduction/oxidation reactions. **Figure** **3**a,b illustrate temporal photocatalytic H_2_ and O_2_ evolution half‐reactions of LaTaON_2_, La_0.1_Ca_0.9_TaO_1+_ *_y_*N_2‐_ *_y_* (*x* = 0.9) and CaTaO_2_N under visible light illumination (*λ* ≥ 420 nm). Complete results for all solid solutions can be found in Figure S11, Supporting Information. Here, sodium sulfite and silver nitrate aqueous solution are used as sacrificial agents to promote H_2_ and O_2_ evolution half‐reactions, respectively. In both cases, La_0.1_Ca_0.9_TaO_1+_ *_y_*N_2‐_ *_y_* (*x* = 0.9) shows the highest activity with striking improvements over its parent compounds LaTaON_2_ and CaTaO_2_N for H_2_ and O_2_ evolutions. In particular, LaTaON_2_ and CaTaO_2_N show very low photocatalytic O_2_ evolution rate which is accelerated by more than one order of magnitude in La_0.1_Ca_0.9_TaO_1+_ *_y_*N_2‐_ *_y_* (*x* = 0.9). Water oxidation into O_2_ is four‐proton‐four‐electron reaction that is considered as the rate‐limited step for overall water splitting reactions.^[^ ^41^ ^]^ The realization of both O_2_ and H_2_ evolution half‐reactions with La_0.1_Ca_0.9_TaO_1+_ *_y_*N_2‐_ *_y_* (*x* = 0.9) opens the avenue toward overall water splitting reaction.

**Figure 3:**
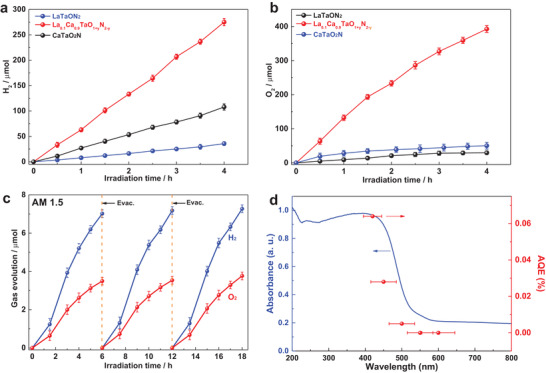
a) Photocatalytic H_2_ evolution for LaTaON_2_, La_0.1_Ca_0.9_TaO_1+_ *_y_*N_2‐_ *_y_* (*x* = 0.9) and CaTaO_2_N under visible light illumination (*λ* ≥ 420 nm). Sodium sulfite aqueous solution (0.05 m) was used as sacrificial agent and 1 wt% Pt as reductive cocatalyst was loaded onto sample powders. b) Photocatalytic O_2_ evolution for LaTaON_2_, La_0.1_Ca_0.9_TaO_1+_ *_y_*N_2‐_ *_y_* (*x* = 0.9) and CaTaO_2_N under visible light illumination (*λ* ≥ 420 nm). Silver nitrate aqueous solution (0.05 m) was applied as sacrificial agent and 2 wt% CoO_x_ as oxidative cocatalyst was loaded onto sample powders. 0.2 g La_2_O_3_ was added to maintain the pH at ≈8.5. c) Direct water splitting reactions for La_0.1_Ca_0.9_TaO_1+_ *_y_*N_2‐_ *_y_* (*x* = 0.9) under AM 1.5 illumination, evacuation was performed every 6 h and 0.5 wt% RhCrO*_x_* as a cocatalyst was loaded onto sample powders. d) Action spectra of La_0.1_Ca_0.9_TaO_1+_ *_y_*N_2‐_ *_y_* (*x* = 0.9) for direct water splitting.

Prior to overall water splitting testing, the activity of La_0.1_Ca_0.9_TaO_1+_ *_y_*N_2‐_ *_y_* (*x* = 0.9) was further investigated by varying the amount of cocatalysts loaded. 1 wt% Pt and 3 wt% CoO*_x_* were found to be the optimal loading amount for H_2_ and O_2_ evolution half‐reactions (Figure S12a,b, Supporting Information), respectively. An apparent quantum efficiency (AQE) as high as 9.4% at 420 ± 20 nm has been achieved for O_2_ evolution half‐reactions which stands as the highest value reported to date on LaTaON_2_ and CaTaO_2_N. Detailed comparison of our materials with other metal oxynitrides in the literatures is given in Table S3, Supporting Information. La_0.1_Ca_0.9_TaO_1+_ *_y_*N_2‐_ *_y_* (*x* = 0.9) is one of few compounds active for both photocatalytic water reduction and oxidation reactions under visible light illumination. Moreover, its activity is apparently better than or at least comparable to other metal oxynitrides reported.

Nevertheless, our attempts for overall water splitting using both Pt and CoO*_x_* loaded La_0.1_Ca_0.9_TaO_1+_ *_y_*N_2‐_ *_y_* (*x* = 0.9) did not produce stoichiometric H_2_ and O_2_. This is probably due to the drawback of Pt cocatalyst that promotes back reactions on the products.^[^ ^42^ ^]^ Thereby, RhCrO*_x_* was loaded onto La_0.1_Ca_0.9_TaO_1+_ *_y_*N_2‐_ *_y_* (*x* = 0.9) as a cocatalyst which is known to be resistant to the back reactions.^[^ ^43^, ^44^ ^]^ In this case, successful overall water splitting with a H_2_/O_2_ molar ratio of 2:1 was achieved both under visible light (*λ* ≥ 420 nm) and simulated AM 1.5 illuminations (Figure 3d, Figure S12c,d, Supporting Information) while no activity was observed for LaTaON_2_ and CaTaO_2_N. Although CaTaO_2_N has been reported to be active for overall water splitting,^[^ ^45^ ^]^ as far as comparing the samples with La_0.1_Ca_0.9_TaO_1+_ *_y_*N_2‐_ *_y_* (*x* = 0.9) and CaTaO_2_N prepared in the present experiments, La_0.1_Ca_0.9_TaO_1+_ *_y_*N_2‐_ *_y_* (*x* = 0.9) showed much higher activity. The activity for overall water splitting was further investigated for other compositions particularly those near *x* = 0.9. The activity shows a clear dependence on the composition and reaches the highest value at *x* = 0.9 (Figure S12c, Supporting Information). As all samples were prepared under the same conditions, such strong correlations between composition and activity suggest that some intrinsic factors probably play an important role here. One possible factor is the Ta—O/N—Ta bond angle that changes along with composition and is critical in shaping the electronic structure. This can be inferred from their conduction band edge positions which are very sensitive to Ta—O/N—Ta bond angle. As already seen previously, Ta—O/N—Ta bond angle likely reaches an optimal point at *x* = 0.9 whereby band edge positions are properly adjusted so that a high driving force of ≥ 0.5 V is available for both water reduction and oxidation reactions. The overall water splitting of La_0.1_Ca_0.9_TaO_1+_ *_y_*N_2‐_ *_y_* (*x* = 0.9) was further investigated by changing the wavelength of incident photons. The action spectra for overall water splitting at *x* = 0.9 are illustrated in Figure 3d. TheAQE shows a clear dependence on incident photon wavelength and matches well with the absorption spectra, confirming real photon‐driven processes. More importantly, the activity of La_0.1_Ca_0.9_TaO_1+_ *_y_*N_2‐_ *_y_* (*x* = 0.9) is maintained for extended cycles of usage, indicating a good stability. The sample powders after these experiments show identical XRD patterns to those before the photocatalytic measurement experiments and STEM‐EDX mapping suggests a homogeneous distribution of all constituent elements (Figure S13, Supporting Information), suggesting the good stability nature of the samples under water splitting conditions.

To gain a deep insight into the origin of the improved photocatalytic activity, we have performed photoelectrochemical (PEC) analysis on LaTaON_2_, La_0.1_Ca_0.9_TaO_1+_ *_y_*N_2‐_ *_y_* (*x* = 0.9) and CaTaO_2_N. La_0.1_Ca_0.9_TaO_1+_ *_y_*N_2‐_ *_y_* (*x* = 0.9) exhibits a higher anodic photocurrent at the bias of over 0.5 V (**Figure** **4**a,b) and smaller interfacial charge transfer resistance than LaTaON_2_ and CaTaO_2_N under both visible light (*λ* ≥ 420 nm) and simulated AM 1.5 illuminations (Figure S14, Supporting Information), indicating a better charge separation capability in the solid solution. These PEC results are consistent with the photocatalytic results. This deduction is also supported by time‐resolved photoluminescence (PL) decay spectra (Figure 4c). La_0.1_Ca_0.9_TaO_1+_ *_y_*N_2‐_ *_y_* (*x* = 0.9) gives a much slower PL decay compared with LaTaON_2_ and CaTaO_2_N. Specifically, its average PL lifetime is extended up to 50.41 ns while LaTaON_2_ and CaTaO_2_N have the much shorter lifetime of 32.44 and 5.53 ns, respectively (Table S4, Supporting Information). This confirms that behavior of photo‐generated charges can be modified by forming solid solutions. The behavior of photo‐generated electrons was further investigated by open‐circuit voltage (*V*_oc_) decay (OCVD) measurements in which O_2_ was used as an electron scavenger and was compared with control experiment using inert Ar (Figure 4d). OCVD records the dissipation processes of photo‐generated electrons which are stored in semiconductors during light illumination.^[^ ^46^ ^]^ O_2_ is a useful prober to those electrons that reach surface and are of chemical reactivity.^[^ ^33^ ^]^ For La_0.1_Ca_0.9_TaO_1+_ *_y_*N_2‐_ *_y_* (*x* = 0.9), O_2_ considerably accelerates OCVD decay with respect to Ar, indicating that a large amount of photo‐generated electrons are accessible to sample surface for chemical reactions. On the contrary, LaTaON_2_ and CaTaO_2_N show little changes on OCVD decay profiles in O_2_ and Ar, implying that most photo‐generated electrons are consumed by recombining with holes or trapping at surface rather than participate in chemical reactions.

**Figure 4:**
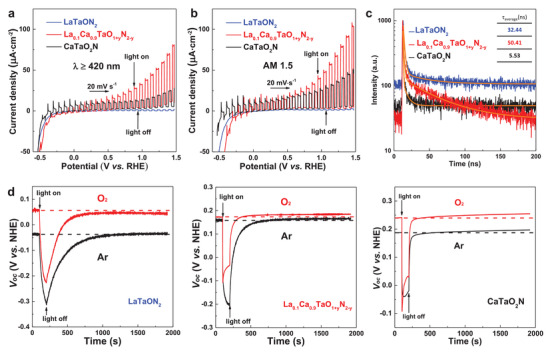
Linear sweep voltammetry of LaTaON_2_, La_0.1_Ca_0.9_TaO_1+_ *_y_*N_2‐_ *_y_* (*x* = 0.9) and CaTaO_2_N under chopped a) visible light illumination (*λ* ≥ 420 nm) and b) simulated AM 1.5 illumination. c) Time‐resolved photoluminescence decay spectra of LaTaON_2_, La_0.1_Ca_0.9_TaO_1+_ *_y_*N_2‐_ *_y_* (*x* = 0.9) and CaTaO_2_N monitored at 625 nm by time‐correlated single‐photon counting. 471.8 nm light generated by a picosecond pulsed light‐emitting diode was used to excite the samples. Average lifetime is tabulated in inset table. d) Open‐circuit voltage (*V*_oc_) decay (OCVD) profiles of photoelectrodes fabricated from LaTaON_2_, La_0.1_Ca_0.9_TaO_1+_ *_y_*N_2‐_ *_y_* (*x* = 0.9) and CaTaO_2_N powders in either Ar or O_2_ atmosphere. The illumination initiated once *V*_oc_ was stabilized in the dark and was terminated at 100 s. *V*_oc_ in the dark was marked by dotted line.

In summary, overall water splitting with stoichiometric H_2_/O_2_ ratio under visible light (*λ* ≥ 420 nm) has been achieved in solid solutions between LaTaON_2_ and CaTaO_2_N, both of which are inactive photocatalysts under the same conditions. The optimal constituent compositions of La_0.1_Ca_0.9_TaO_1+_ *_y_*N_2‐_ *_y_* (*x* = 0.9) shows the highest activity with a good durability and stability. The modulated key parameters including Ta—O/N bond length, Ta—O/N—Ta bond angle, defects concentration, bandgap, and band edge alignments greatly promote the separation of photogenerated charges as evidenced by the combination of several characterizations. Moreover, the appropriate band edges guarantee the sufficient driving force of the charge carriers for both water reduction and oxidation half‐reactions. The global merits of the developed solid solution make it a promising solar‐driven photocatalyst for overall water splitting.

## Conflict of Interest

The authors declare no conflict of interest.

## Supporting information

Supporting Information
