# Supplementary material for: Perovskite Oxynitride Solid Solutions of LaTaON2‐CaTaO2N with Greatly Enhanced Photogenerated Charge Separation for Solar‐Driven Overall Water Splitting
Source: Adv Sci (Weinh). 2020 Nov 25;8(2):2003343. doi: 10.1002/advs.202003343 (PMC7816695; doi:10.1002/advs.202003343)
Supplement: Supplementary file 1 — Supporting Information [file ADVS-8-2003343-s001.pdf]

© 2020 Wiley-VCH GmbH

ADVANCED SCIENCE
Open Access

# Supporting Information

for *Adv. Sci.*, DOI: 10.1002/advs.202003343

Perovskite Oxynitride Solid Solutions of $LaTaON_2$-$CaTaO_2N$ with Greatly Enhanced Photogenerated Charge Separation for Solar-driven Overall Water Splitting

*Yawei Wang, Yuyang Kang, Huaze Zhu, Gang Liu*, John T. S. Irvine* and Xiaoxiang Xu**

# Supporting Information

## Perovskite oxynitride solid solutions of $LaTaON_2$-$CaTaO_2N$ with greatly enhanced photogenerated charge separation for solar-driven overall water splitting

*Yawei Wang, Yuyang Kang, Huaze Zhu, Gang Liu*, John T. S. Irvine* and Xiaoxiang Xu**

*Materials synthesis*: Perovskite oxynitride solid solutions $LaTaON_2$-$CaTaO_2N$, i.e. $La_{1-x}Ca_xTaO_{1+y}N_{2-y}$ ($0 \leq x, y \leq 1$), were synthesized by ammonolysis of proper metal oxide precursors. These precursors were synthesized *via* a sol-gel method: typically, for the synthesis of precursors for $La_{0.1}Ca_{0.9}TaO_{1+y}N_{2-y}$ (x = 0.9), 6.75 mL acetic acid (Aladdin, 99.9%) and 0.75 mL acetic anhydride (Aladdin, 99.9%) were mixed up to form a transparent solution. Appropriate amounts of $Ca(NO_3)_2{\cdot}4H_2O$ (Aladdin, 99.9%), $La(NO_3)_3{\cdot}6H_2O$ (Aladdin, 99.9%) and $TaCl_5$ (Aladdin, 99.99%) ethanol solution (0.2 M) were added to above solution under magnetic stirring. After completely dissolving all chemicals, the solution was heated in an oven at 323 K for 24 h to allow slow hydrolysis and precipitation. The resultant white precipitants were dried in an oven at 323 K for 12 h, calcined in $N_2$ at 523 K for 3 h and calcined again in $O_2$ at 873 K for another 5 h. The final white powders were then ground and used as metal oxide precursors for further ammonolysis.

Ammonolysis of metal oxide precursors was carried out in a tube furnace at high temperatures under flowing ammonia gas. Typically, 0.2 g of metal oxide precursors were placed in an alumina boat and were mounted in the tube furnace. Ultrapure ammonia gas (Jiaya Chemical, 99.999%) was directed into the tube furnace with a flow rate of ~ 250 mL$\cdot$ min$^{-1}$. The temperature of the tube furnace was then ramped to 1223 K with a heating rate of 10 K$\cdot$ min$^{-1}$. The furnace was kept at this temperature for 10 h and was cooled to room temperature naturally. The resultant sample powders were washed with diluted hydrochloric acid for three times, distilled water for five times and dried in an oven at 353 K overnight.

*Materials characterization*: The as-prepared sample powders were examined by X-ray powder diffraction (XRD) techniques on a Bruker D8 Focus diffractometer (Bruker, Germany). Incident radiation was Cu $K_{\alpha1}$ radiation (λ = 1.5405 Å) and $CuK_{\alpha2}$ radiation (λ = 1.5444 Å) with ratio of 2 to 1. Step size and duration for data collection were 0.01° and 10 s. Rietveld refinement was performed on collected data using General Structure Analysis System (GSAS) software package[47]. A pseudo-Voigt function was used as profile function and a Chebyshev polynomial of the first kind was adopted for background fit. Raman spectra were collected on an InVia DXR Raman spectrometer using He-Cd laser with excitation wavelength 514 nm. Optical absorption spectra were collected on a UV-Vis spectrophotometer (JASCO-750, Japan). The spectrophotometer was operated in a diffuse reflectance mode with an integrating sphere. The reference non-absorbing material is $BaSO_4$. A field emission scanning electron microscope (FESEM, Hitachi S4800, Japan), a transmission electron microscope (TEM, JEOL-2100, Japan) and a scanning transmission electron microscope (STEM, Titan$^3$ G2 60-300, USA) were used to examine the microstructures of sample powders. Thermogravimetric analysis (TGA) of sample powders was performed on a Labsysevo system (SETRAM, France) in flowing air. A constant heating and cooling rate of 10 $K \cdot min^{-1}$ was applied between room temperature and 1473 K. Nitrogen adsorption-desorption isotherms of sample powders were analyzed on NOVA 2200e adsorption apparatus (Quantachrome, USA). Specific surface area and pore-size distribution of sample powders were calculated based on the Brunauer-Emmett-Teller (BET) model and Barrett-Joyner-Halenda (BJH) model, respectively. Surface conditions of sample powders were inspected by X-ray photoelectron spectroscopy (XPS) on Thermo Escalab 250 with a monochromatic Al $K_{\alpha}$ source (1486.6 eV). The binding energy of core-level electrons was adjusted according to C 1s peak at 284.7 eV which belongs to adventitious carbon[48]. The XPS data were fitted and analyzed by XPS PEAKFIT software using Gaussian-Lorentzian function (Lorentzian weighting of 20%) for profile fit and Shirley type backgrounds.

*Photocatalytic water splitting*: Photocatalytic performance of sample powders was evaluated by monitoring their hydrogen and/or oxygen evolution under visible light ($\lambda \geq 420$ nm) or simulated AM 1.5 illuminations. These experiments were carried out in a top-illumination-type glass reactor with a quartz window on the top. The reactor was connected to a closed gas circulation and evacuation system (Perfect Light, Labsolar-IIIAG, China) (**Figure S10**). Sacrificial agents were used when water splitting half reactions were studied. Sodium sulfite or silver nitrate aqueous solution (0.05 M) was applied as sacrificial agent to promote water reduction or oxidation reactions. Proper cocatalysts were also used to further facilitate water reduction or oxidation reactions. For water reduction reactions, Pt was introduced as a cocatalyst loaded onto sample powders by a thermal deposition method[49]: sample powders were impregnated with appropriate amounts of $H_2PtCl_6$ aqueous solution to form slurry. The slurry was heated at 353 K on a hot plate until dry and further calcined in an oven at 573 K. For oxygen evolution reaction, $CoO_x$ was introduced as a cocatalyst deposited onto sample powders according to previous reports[33, 50]: sample powders were immersed into proper amounts of cobalt nitrate aqueous solution under ultrasonic bath. The resultant slurry was heated on a hot plate until dry, calcined in an $NH_3$ atmosphere with a flow of ~ 200 mL· min$^{-1}$ at 1023 K for 1 h and calcined again in air at 423 K for another 1 h. For direct water splitting reactions, $RhCrO_x$ was used as a cocatalyst loaded onto sample powders according to literatures[44, 51]: sample powders were dispersed ultrasonically in methanol which was then immersed into proper amounts of $RhCl_3 \cdot 3H_2O$ (Aladdin, 99.9%) and $Cr(NO_3)_3 \cdot 9H_2O$ (Aladdin, 99.9%) aqueous solution. The resultant solution was dried in an oven and calcined at 623 K under $N_2$ (flow rate ~ 100 mL min$^{-1}$) for 1 h. In a typical photocatalytic experiment, 100 mg sample powders loaded with proper amounts of cocatalysts were placed in the reactor containing 100 mL aqueous solution with or without sacrificial agent. 0.2 g $La_2O_3$ (Aladdin, 99.9%) was introduced to control the pH at ca. 8.5 during water oxidation reactions[52]. The reactor was evacuated for 40 min to remove gas dissolved in the solution. A 300 W Xeon

lamp (Perfect Light, PLX-SXE300, China) was used as the light source. Visible light or AM 1.5 illumination was generated by filtering the output of the lamp using a UV cutoff filter ($\lambda \geq$ 420 nm) or a AM 1.5 filter. The gas component within the reactor was analyzed by an on-line gas chromatograph (TECHCOMP, GC7900, China) equipped with a thermal conductivity detector and 5 Å molecular sieve columns. Ar was used as a carrier gas. For the determination of apparent quantum efficiency (AQE), band pass filters at 420 nm, 450 nm, 500 nm, 550 nm and 600 nm were applied to the Xeon lamp to produce monochromatic light. The photon flux at individual wavelength was gauged using a quantum meter (Apogee MP-300, China) (**Table S5**). AQE at each monochromatic wavelength was evaluated according to following formula[7]:

$$\text{Apparent quantum efficiency (AQE)} = \frac{\text{n} \times \text{moles of gas production per hour}}{\text{moles of photon flux per hour}} \times 100\% \quad (1)$$

where n = 2 for hydrogen production and 4 for oxygen production.

*Photoelectrochemical (PEC) measurement*: Photoelectrodes deposited with sample powders were used for PEC measurement. These photoelectrodes were fabricated by electrophoretic deposition method[53]: two pieces of fluorine doped tin oxide glass (FTO) (1 cm × 3 cm) were immersed into 40 mL acetone containing 40 mg sample powders and 10 mg iodine (Sigma-Aldrich, ≥ 99.8%). The FTO glasses were kept in parallel with a separation of 1 cm and their conductive side facing inward. A constant electrical bias ~ 10 V was applied between the glasses for 3 min under potentiostatic control (Keithley 2450 Source Meter, USA). FTO glass at the anode side was quickly deposited with sample powders (deposition area ~ 1 $cm^2$) and was used as the photoelectrode. The photoelectrode was calcined at 673 K for 1 h for the

removal of iodine absorbed. A few drops of $TaCl_5$ (Alfa-Aesar, 99.9%) methanol solution (10 mM) were introduced into the photoelectrode which was dried at 623 K for 15 min. After repeating this procedure for five times, the photoelectrode was calcined at 673 K in ammonia (flow rate ~ 200 mL· $min^{-1}$) for 1 h to strengthen particle interconnections. PEC analysis was performed in a three-electrode configuration, whereby photoelectrode, Pt foil (1 cm × 1 cm) and Ag/AgCl electrode were used as the working, counter and reference electrodes, respectively. The analysis was controlled by a Zahner electrochemical workstation. $K_3PO_4/K_2HPO_4$ (0.1 M) was used as electrolyte and buffer (pH = 13). A 300 W Xeon lamp (Perfect Light, PLX-SXE300, China) equipped with a UV cutoff filter ($\lambda \geq 400$ nm) or AM1.5 filter was used as the light source. The light beam was rectified by an electronic timer and shutter (DAHENG, GCI-73, China). Impedance spectra were collected under proper bias with an AC signal from $10^5$ Hz to $10^{-1}$ Hz with 10 mV amplitude. Impedance data at 1000 Hz was adopted to extract capacitance data which were used to perform Mott-Schottky (MS) analysis.

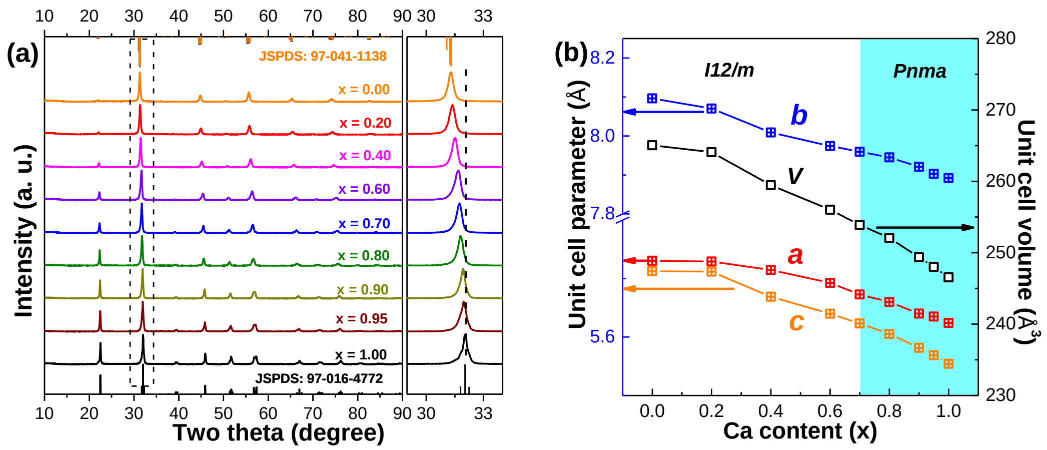


**Figure S1** (a) X-ray powder diffraction patterns of $La_{1-x}Ca_xTaO_{1+y}N_{2-y}$ ($0 \leq x, y \leq 1$); (b) refined unit cell parameters as a function of Ca content (x); structures with different space group are marked by shaded area

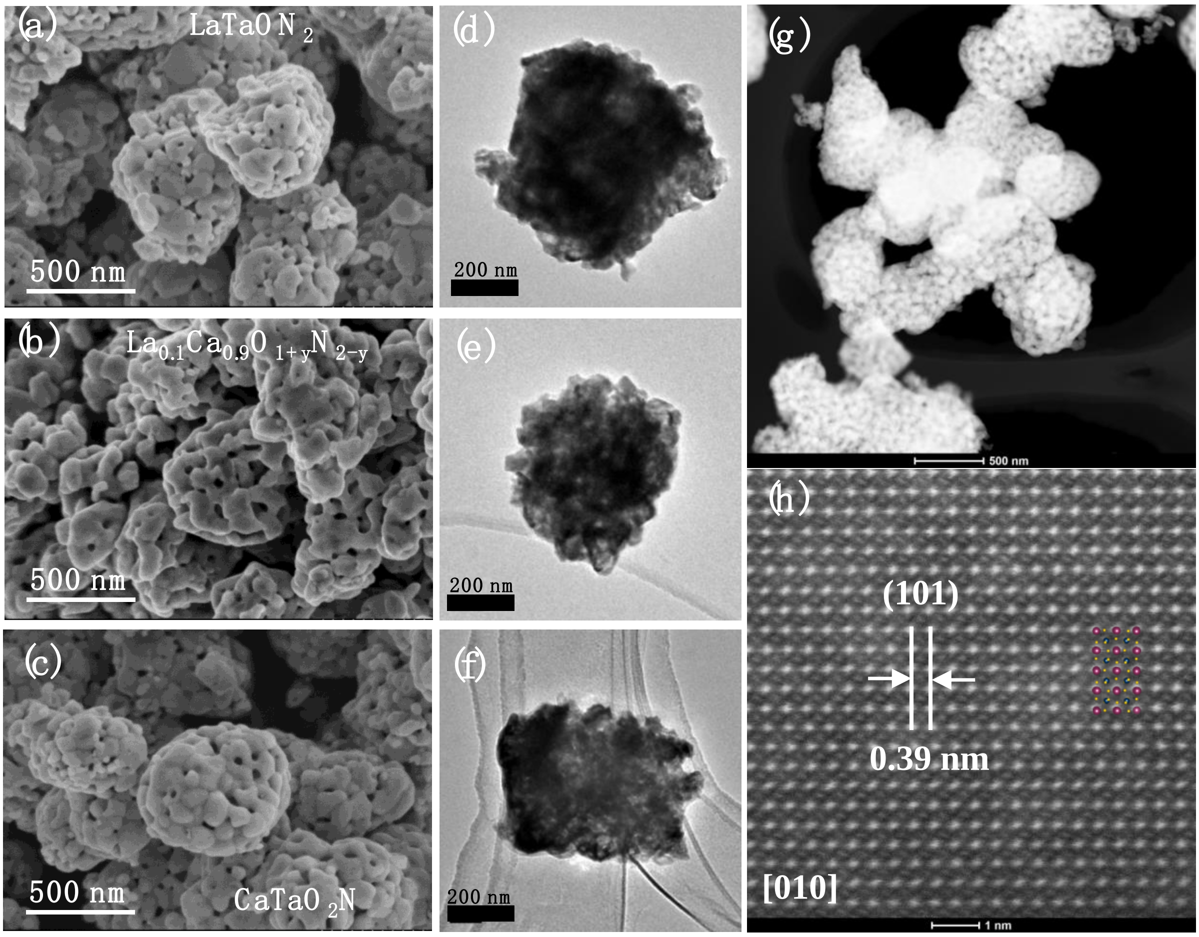


**Figure S2** Field emission scanning electron microscopic images (a, b and c) and transmission electron microscopic images (d, e and f) for $LaTaON_2$, $La_{0.1}Ca_{0.9}TaO_{1+y}N_{2-y}$ (x = 0.9) and $CaTaO_2N$, respectively; dark-field scanning transmission electron microscopy image (g) and high-angle annular dark-field scanning transmission electron microscopy (HAADF-STEM) image (h) of $La_{0.1}Ca_{0.9}TaO_{1+y}N_{2-y}$ (x = 0.9). HAADF-STEM was taken along [010] direction. Marked fringe corresponds to (101) plane. Depicted crystal structure of $La_{0.1}Ca_{0.9}TaO_{1+y}N_{2-y}$ (x = 0.9) projected along [010] direction was also inserted.

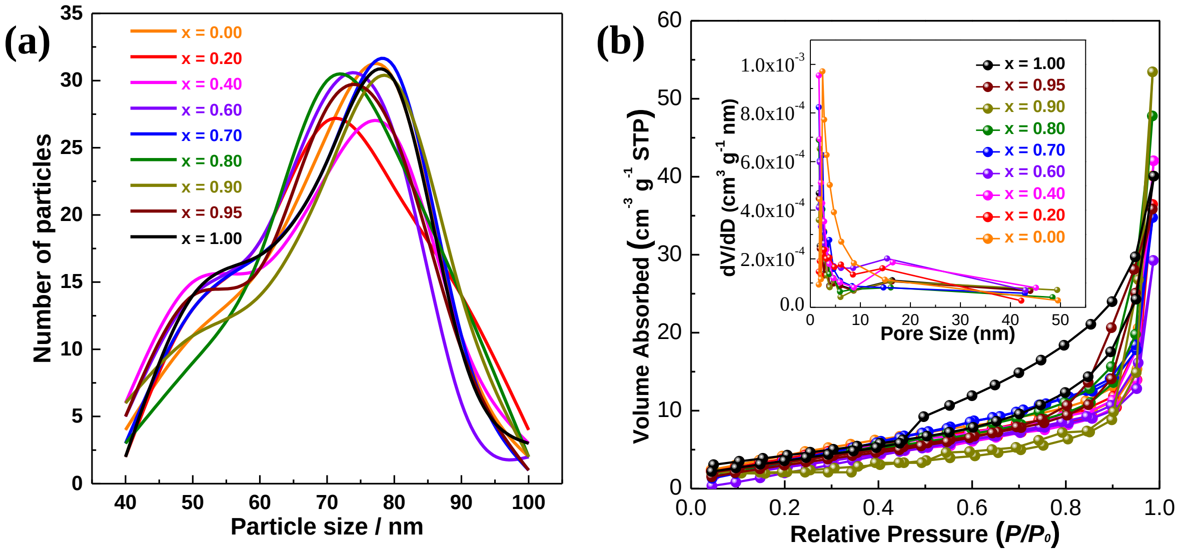


**Figure S3** (a) Particle size distribution of $La_{1-x}Ca_xTaO_{1+y}N_{2-y}$ ($0 \leq x, y \leq 1$); (b) BET surface analysis of $La_{1-x}Ca_xTaO_{1+y}N_{2-y}$ ($0 \leq x, y \leq 1$), pore size distributions are shown as inset.

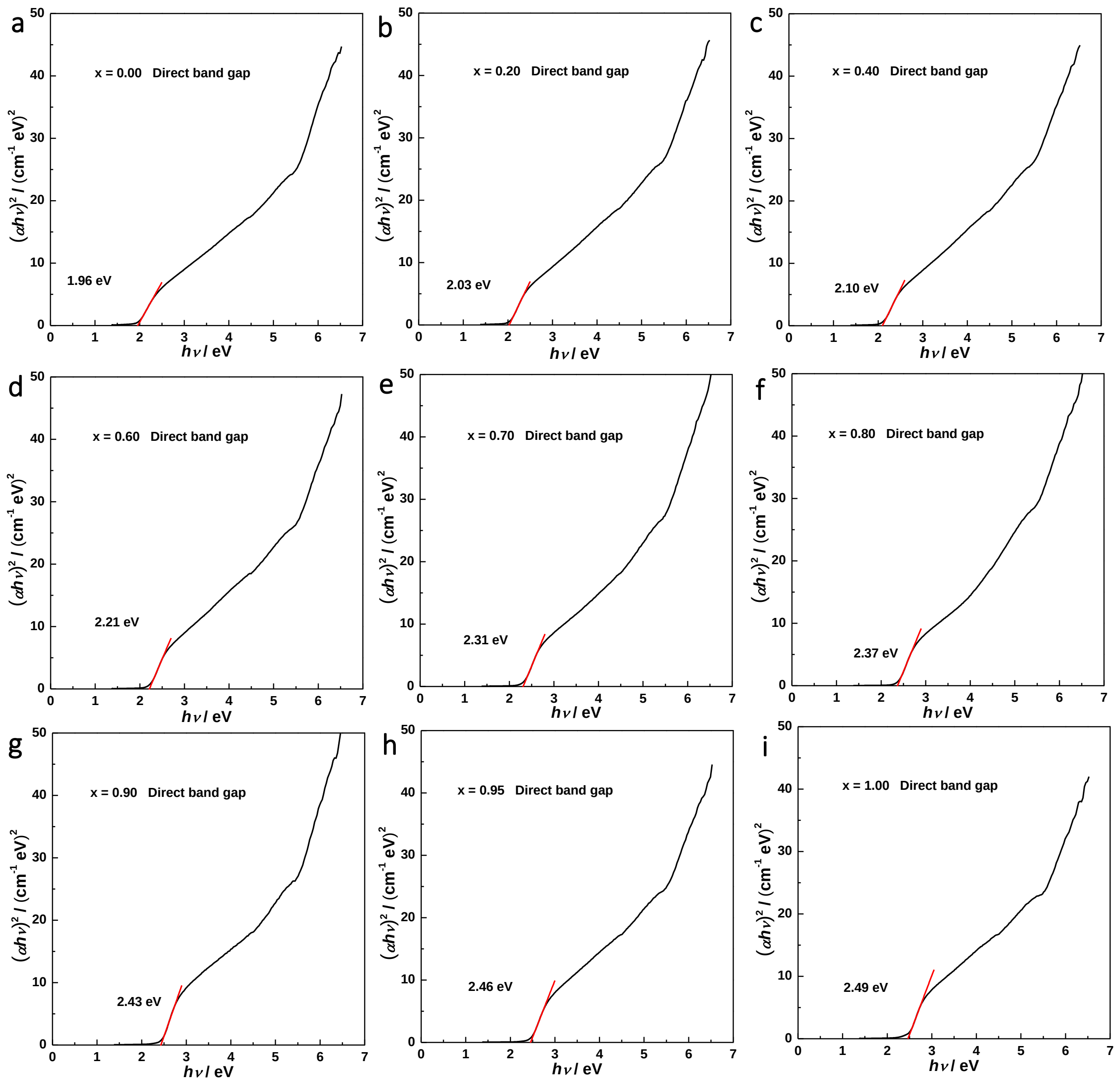


**Figure S4** Tauc plots of $La_{1-x}Ca_xTaO_{1+y}N_{2-y}$ ($0 \leq x, y \leq 1$) for direct band gap determination: (a) x = 0.00; (b) x = 0.20; (c) x = 0.40; (d) x = 0.60; (e) x = 0.70; (f) x = 0.80; (g) x = 0.90; (h) x = 0.95 and (i) x = 1.00.

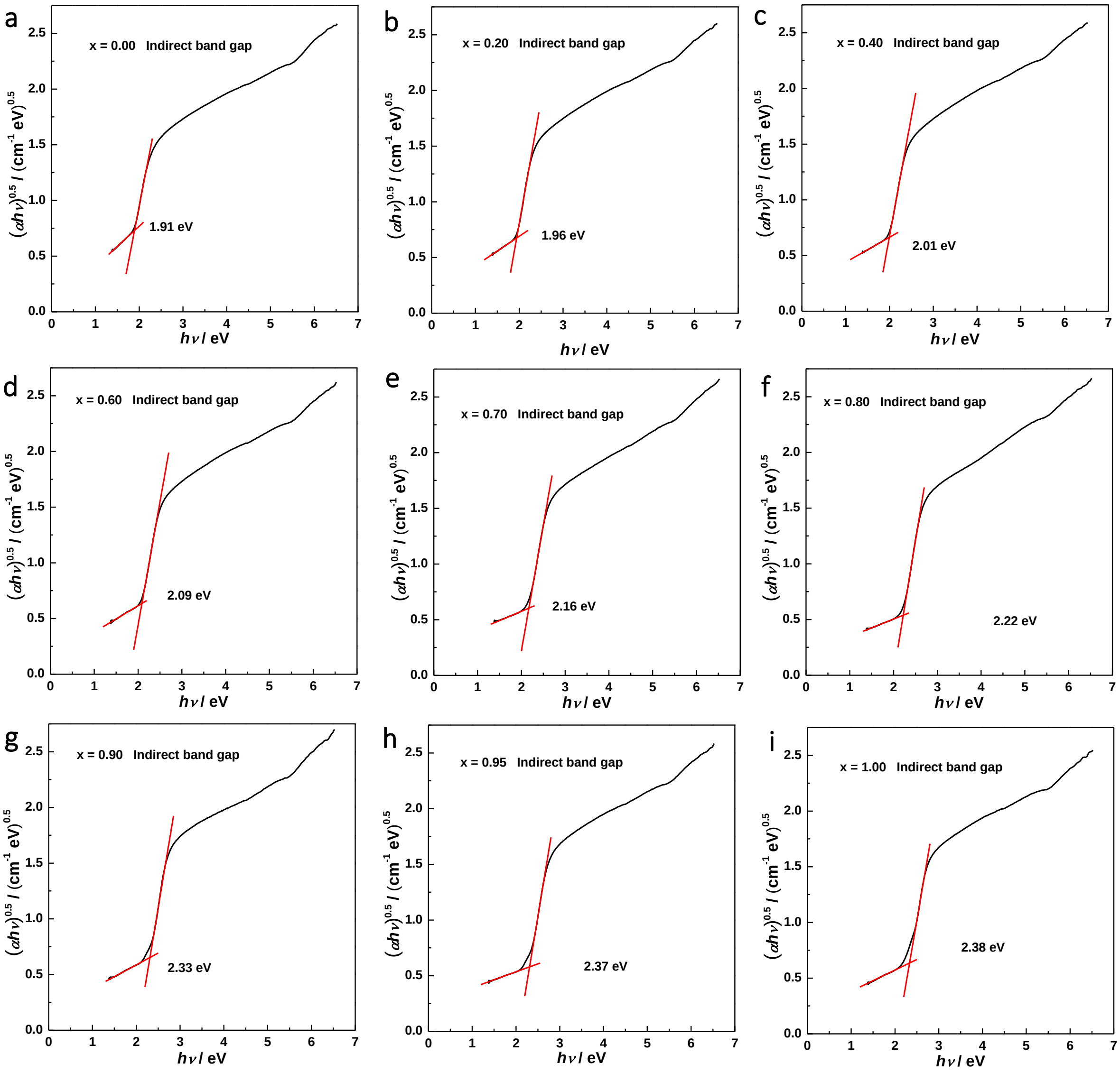


**Figure S5** Tauc plots of $La_{1-x}Ca_xTaO_{1+y}N_{2-y}$ ($0 \leq x, y \leq 1$) for indirect band gap determination: (a) x = 0.00; (b) x = 0.20; (c) x = 0.40; (d) x = 0.60; (e) x = 0.70; (f) x = 0.80; (g) x = 0.90; (h) x = 0.95 and (i) x = 1.00.

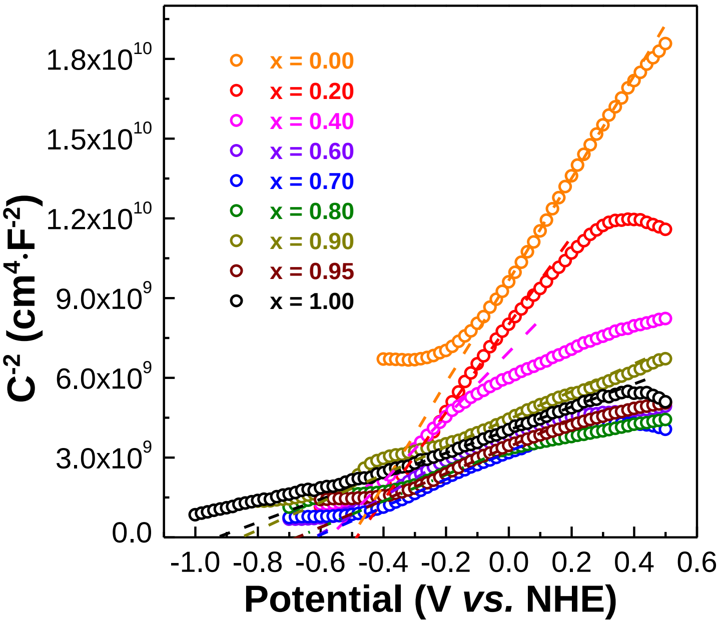


**Figure S6** Mott-Schottky plots of $La_{1-x}Ca_xTaO_{1+y}N_{2-y}$ ($0 \leq x, y \leq 1$), capacitance was extracted from impedance analysis at 1000 Hz with 10 mV amplitude

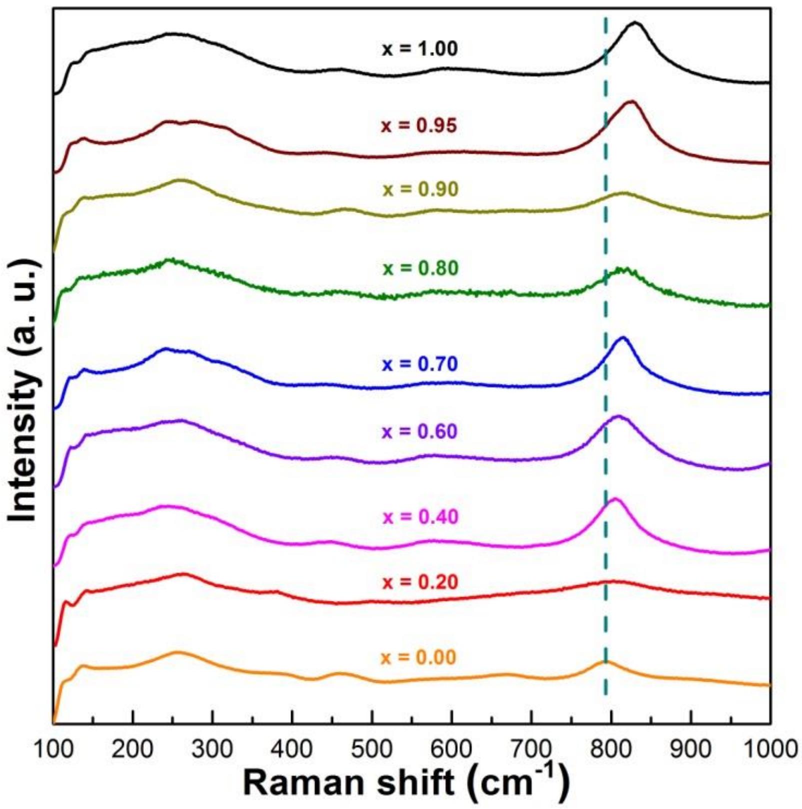


**Figure S7** Raman spectra of $La_{1-x}Ca_xTaO_{1+y}N_{2-y}$ ($0 \leq x, y \leq 1$); dotted line is a guide to the eye.

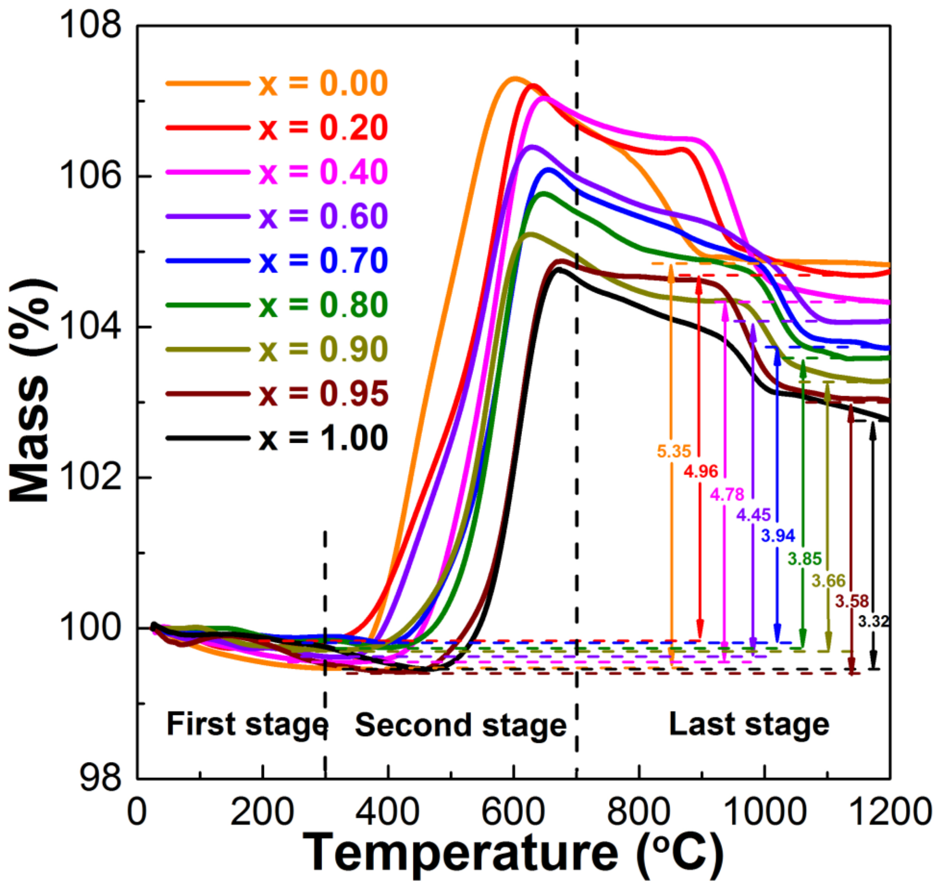


**Figure S8** Thermogravimetric analysis (TGA) of $La_{1-x}Ca_xTaO_{1+y}N_{2-y}$ ($0 \leq x, y \leq 1$) from room temperature to 1200 °C in air; these curves can be divided into three stages: the first stage is from 323 K to 673 K in which desorption of water/$CO_2$ results in slight mass decrease; the second stage is from 673 K to about 973 K where oxidation processes (oxygen uptake) leads in sharp mass increase; the last stage is from 973 K to 1573 K where nitrogen is released from the structure until a mass plateau is approached. Thereby, mass differences between the plateau and the end of the first stage refer to replacements of nitrogen with oxygen in the structure. These values are used to determine nitrogen content and are tabulated in Table S2 for all samples.

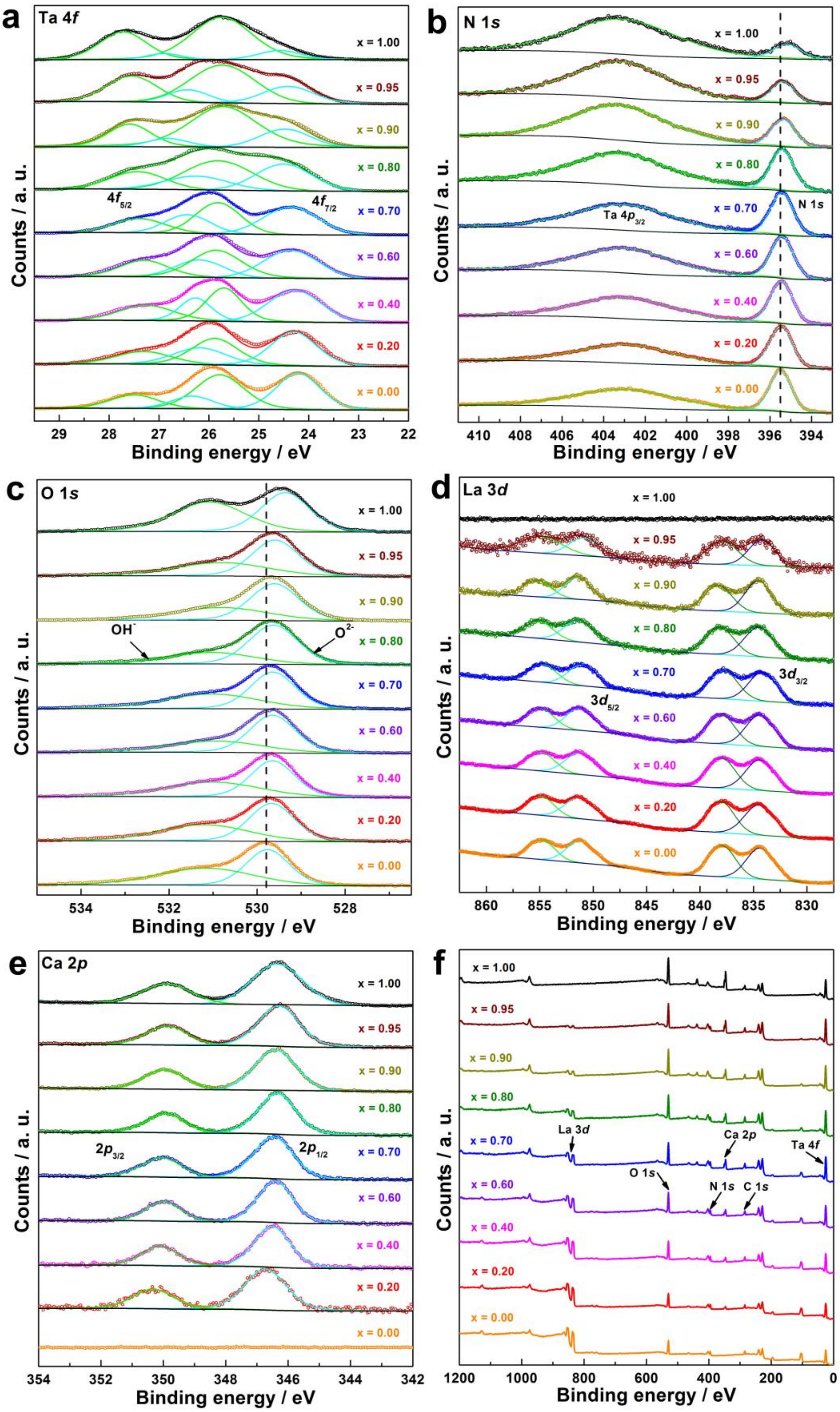


**Figure S9** XPS of $LaTaON_2$, $La_{0.1}Ca_{0.9}TaO_{1+y}N_{2-y}$ (x = 0.9) and $CaTaO_2N$: (a) Ta 4*f* state; (b) N 1*s* state; (c) O 1*s* state; (d) La 3*d* state; (e) Ca 2*p* state and (f) survey spectra.

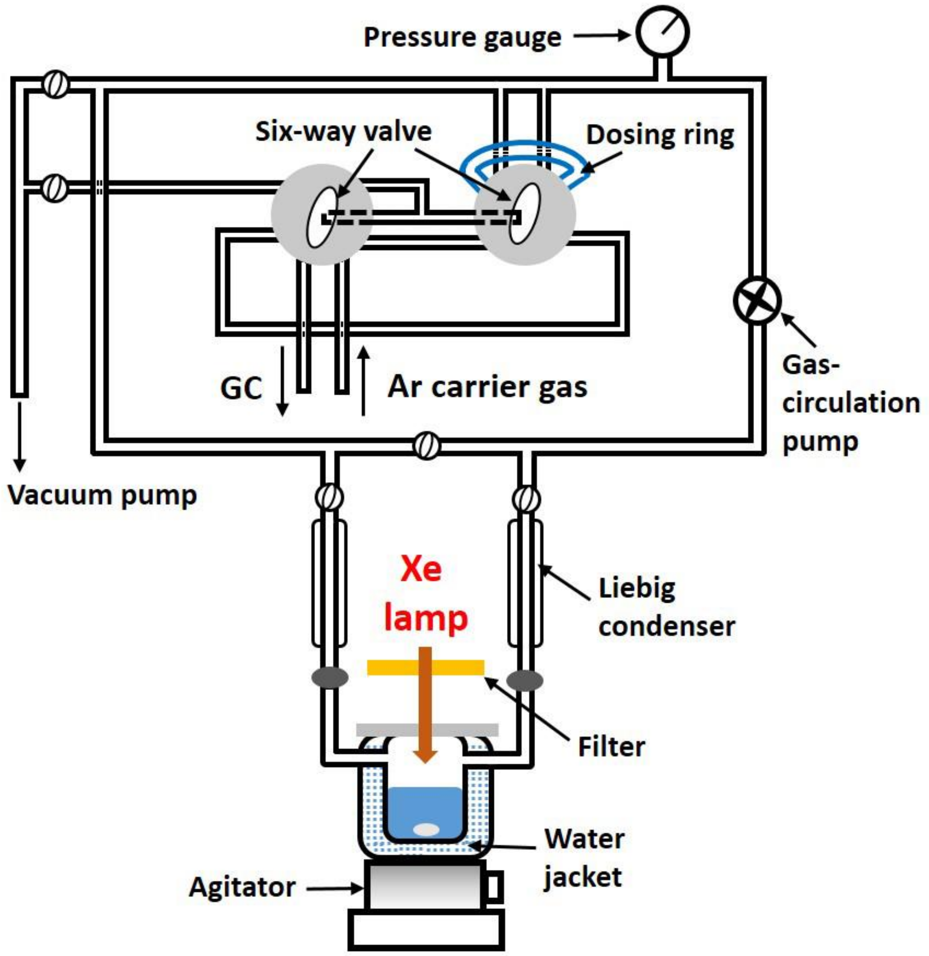


**Figure S10** Schematic representation of experiment setup for photocatalytic water splitting. The aqueous solution containing sample powders is sealed in a glass reactor with a quartz window on the top for light illumination. The reactor is connected to a gas-closed circulation and evacuation system (Perfect Light, Labsolar-IIIAG, China). For direct overall water splitting experiment, gas produced within the reactor was sampled three times for GC measurement at a given point. Data at each point was averaged and standard deviation was analyzed.

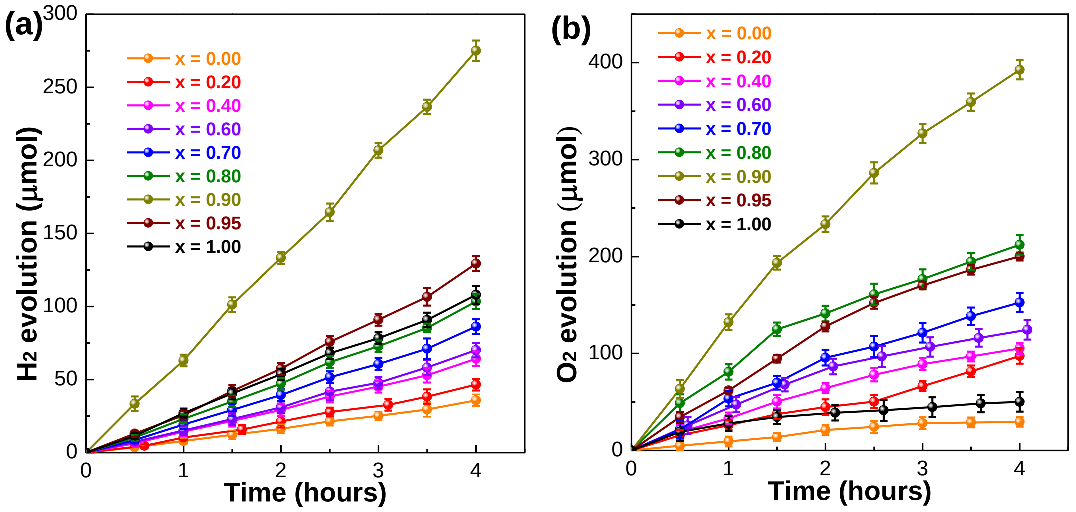


**Figure S11** (a) Photocatalytic $H_2$ evolution for $La_{1-x}Ca_xTaO_{1+y}N_{2-y}$ ($0 \leq x, y \leq 1$) under visible light illumination ($\lambda \geq 420$ nm), sodium sulfite aqueous solution (0.05 M) was used as sacrificial agent and 1 wt% Pt was loaded onto sample powders as a cocatalyst; (b) photocatalytic $O_2$ evolution for $La_{1-x}Ca_xTaO_{1+y}N_{2-y}$ ($0 \leq x, y \leq 1$) under visible light illumination ($\lambda \geq 420$ nm), silver nitrate aqueous solution (0.05 M ) was applied as sacrificial agent and 2 wt% $CoO_x$ was loaded onto sample powders as a cocatalyst, 0.2 g $La_2O_3$ was added to maintain the pH at *ca.* 8.5.

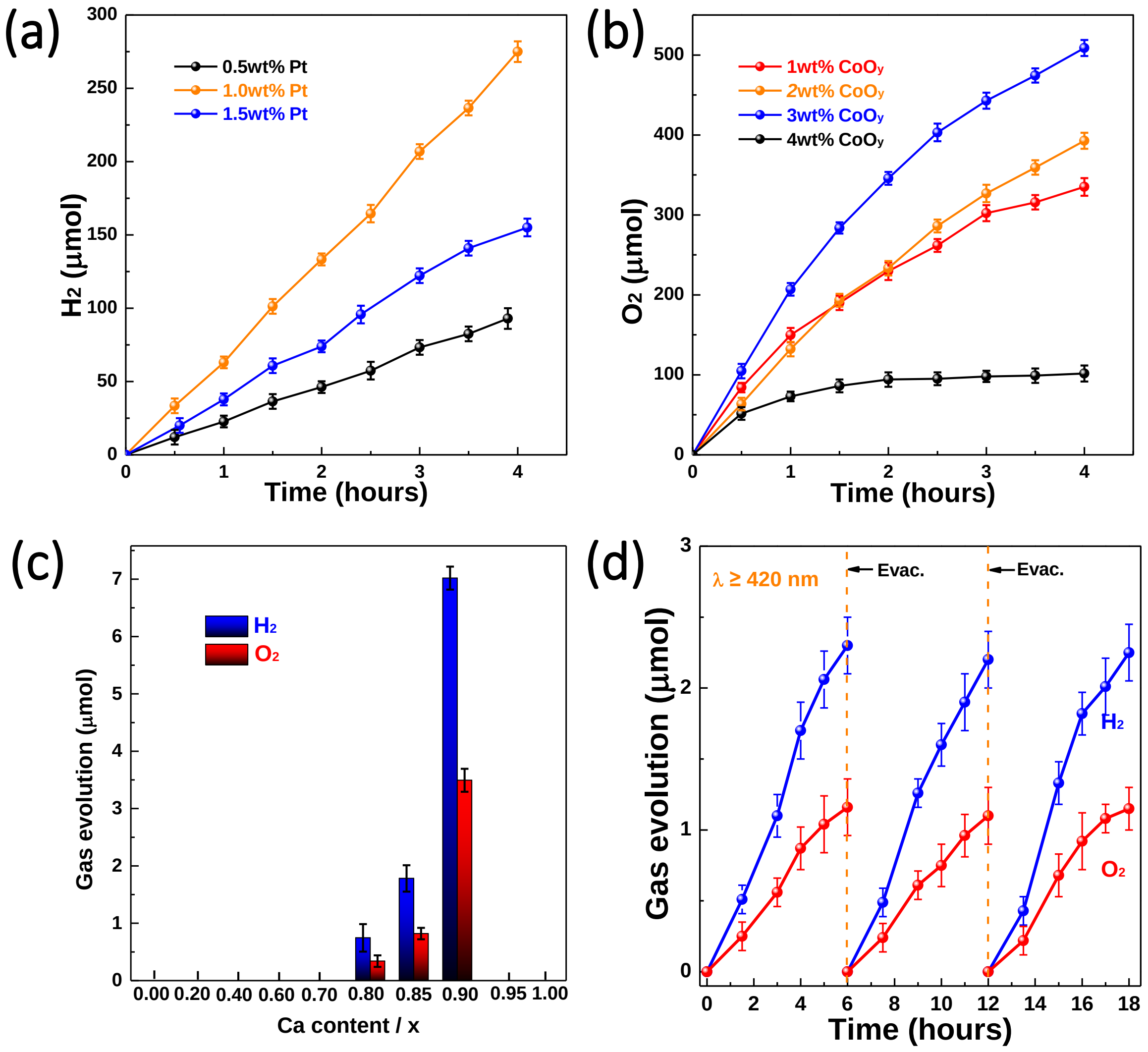


**Figure S12** Photocatalytic $H_2$ evolution for $La_{0.1}Ca_{0.9}TaO_{1+y}N_{2-y}$ (x = 0.9) with different amounts of cocatalyst Pt under visible light illumination (λ ≥ 420 nm), sodium sulfite aqueous solution (0.05 M) was used as sacrificial agent; (b) photocatalytic $O_2$ evolution for $La_{0.1}Ca_{0.9}TaO_{1+y}N_{2-y}$ (x = 0.9) with different amounts of cocatalyst Pt under visible light illumination (λ ≥ 420 nm), silver nitrate aqueous solution (0.05 M) was used as sacrificial agent; (c) histogram of direct water splitting reactions for $La_{1-x}Ca_xTaO_{1+y}N_{2-y}$ (0 ≤ x, y ≤ 1) under AM 1.5 illumination for 6 h, 0.5 wt% $RhCrO_x$ was loaded onto sample powders as a cocatalyst; (d) direct water splitting reactions for $La_{0.1}Ca_{0.9}TaO_{1+y}N_{2-y}$ (x = 0.9) under visible light illumination (λ ≥ 420 nm), evacuation was performed every 6 h and 0.5 wt% $RhCrO_x$ was loaded onto sample powders as a cocatalyst

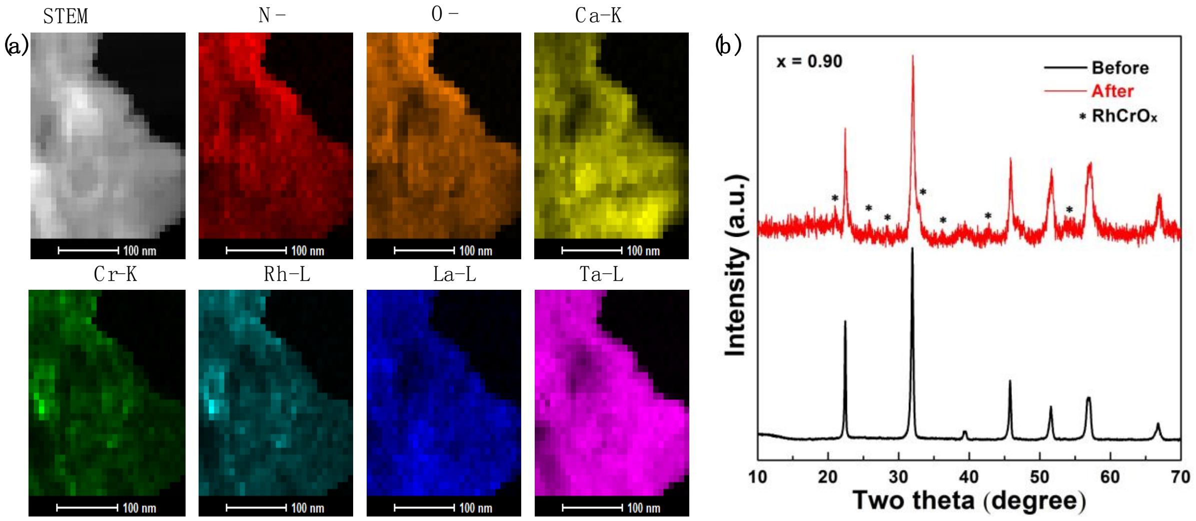


**Figure S13** (a) STEM EDX mapping of $La_{0.1}Ca_{0.9}TaO_{1+y}N_{2-y}$ (x = 0.9) loaded with 0.5 wt% $RhCrO_x$ after overall water splitting reactions; (b) XRD patterns of $La_{0.1}Ca_{0.9}TaO_{1+y}N_{2-y}$ (x = 0.9) before and after photocatalytic water splitting, peaks marked by asterisk correspond to co-catalyst $RhCrO_x$..

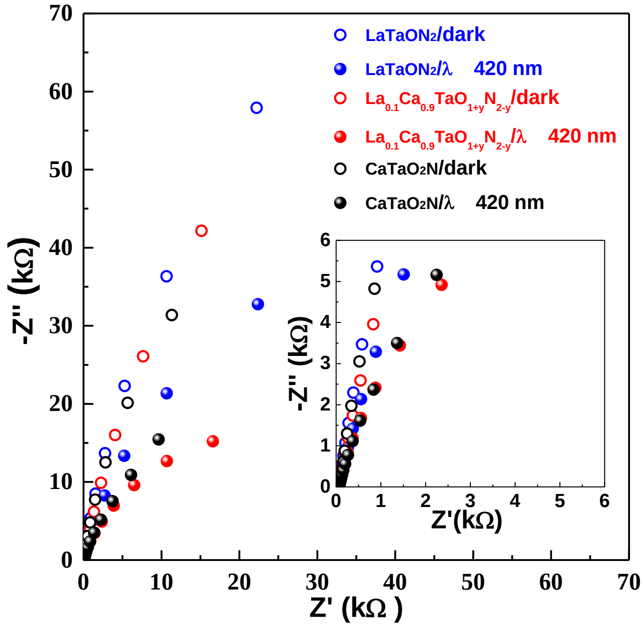


**Figure S14** impedance spectra for $LaTaON_2$, $La_{0.1}Ca_{0.9}TaO_{1+y}N_{2-y}$ (x = 0.9) and $CaTaO_2N$ at open-circuit voltage in dark and under visible light illumination (λ ≥ 420 nm)

**Table S1** Unit cell parameters of as-prepared samples $La_{1-x}Ca_xTaO_{1+y}N_{2-y}$ according to Rietveld refinement, standard deviation is included in the parenthesis.

| x | Space group | *a* (Å) | *b* (Å) | *c* (Å) | α and γ (°) | β (°) | *V* (Å$^3$) | A (°) | D (Å) |
|---|---|---|---|---|---|---|---|---|---|
| 0.00 | *I12/m* | 5.7321(3) | 8.0711(3) | 5.7116(9) | 90.0 | 90.23(3) | 265.05(1) | 166.04(2) | 2.037(3) |
| 0.20 | *I12/m* | 5.7168(3) | 8.0594(9) | 5.7068(3) | 90.0 | 89.91(7) | 262.97(1) | 164.13(3) | 2.035(1) |
| 0.40 | *I12/m* | 5.7145(2) | 8.0126(3) | 5.6693(2) | 90.0 | 89.86(1) | 259.45(6) | 162.83(1) | 2.034(2) |
| 0.60 | *I12/m* | 5.6963(7) | 7.9735(8) | 5.6361(4) | 90.0 | 89.65(2) | 255.91(3) | 160.67(5) | 2.031(1) |
| 0.70 | *I12/m* | 5.2642(4) | 7.9598(2) | 5.6232(6) | 90.0 | 89.53(1) | 253.90(1) | 158.94(2) | 2.029(3) |
| 0.80 | *Pnma* | 5.6601(7) | 7.9452(1) | 5.6052(4) | 90.0 | 90.0 | 252.04(6) | 157.43(1) | 2.028(7) |
| 0.90 | *Pnma* | 5.6394(5) | 7.9210(3) | 5.5836(2) | 90.0 | 90.0 | 249.37(3) | 156.02(3) | 2.026(4) |
| 0.95 | *Pnma* | 5.6351(2) | 7.9031(6) | 5.5689(2) | 90.0 | 90.0 | 248.04(4) | 153.64(4) | 2.024(2) |
| 1.00 | *Pnma* | 5.6234(3) | 7.8918(1) | 5.5538(3) | 90.0 | 90.0 | 246.54(1) | 150.13(2) | 2.023(3) |

A: the average Ta-O/N-Ta bond angle; D: the average Ta-O/N bond length.

**Table S2** Band gap values calculated from Tauc plot, effective chemical formula determined from TGA, and BET specific surface area of as-prepared samples.

| x | Direct band gap (eV) | Indirect band gap (eV) | Effective chemical formula | BET surface area ($m^2\ g^{-1}$) |
|---|---|---|---|---|
| 0.00 | 1.95(1) | 1.91(1) | $LaTaO_{1.28}N_{1.72}$ | 13.7(1) |
| 0.20 | 2.03(4) | 1.96(2) | $Ca_{0.2}La_{0.8}TaO_{1.54}N_{1.46}$ | 12.3(1) |
| 0.40 | 2.15(3) | 2.01(1) | $Ca_{0.4}La_{0.6}TaO_{1.68}N_{1.32}$ | 13.1(1) |
| 0.60 | 2.25(4) | 2.09(1) | $Ca_{0.6}La_{0.4}TaO_{1.88}N_{1.12}$ | 11.1(2) |
| 0.70 | 2.32(1) | 2.16(2) | $Ca_{0.7}La_{0.3}TaO_{2.18}N_{0.82}$ | 14.3(1) |
| 0.80 | 2.38(3) | 2.22(2) | $Ca_{0.8}La_{0.2}TaO_{2.28}N_{0.72}$ | 13.6(1) |
| 0.90 | 2.42(3) | 2.33(1) | $Ca_{0.9}La_{0.1}TaO_{2.32}N_{0.68}$ | 14.9(1) |
| 0.95 | 2.45(3) | 2.37(1) | $Ca_{0.95}La_{0.05}TaO_{2.46}N_{0.54}$ | 13.3(2) |
| 1.00 | 2.47(2) | 2.38(2) | $CaTaO_{2.58}N_{0.42}$ | 15.7(1) |

**Table 3** Metal (oxy)nitride photocatalysts for $H_2$ or $O_2$ evolution from aqueous solutions in the presence of suitable sacrificial reagents.

| Photocatalyst | B.G. | $H_2$ evolution | | $O_2$ evolution | | Ref.(Year) |
|---|---|---|---|---|---|---|
| | | Co-catal. | Acticity (μmol $h^{-1}$)* | Co-catal. | Acticity (μmol $h^{-1}$)* | |
| **$Ca_{0.9}La_{0.1}TaO_{1+y}N_{2-y}$** | **2.42** | **1 wt% Pt** | **62.7** | **3 wt% $CoO_x$** | **207** | **This work** |
| $CaTaO_2N$ | 2.47 | 1 wt% Pt | 27.1 | 2 wt% $CoO_x$ | 27.9 | This work |
| | 2.43 | $RhCrO_y$ | 5.9 | – | 2.9 | [45] (2015) |
| | 2.44 | 1 wt% Pt | 2.5 | – | – | [39] (2020) |
| $LaTaON_2$ | 1.95 | 1 wt% Pt | 7.9 | 2 wt% $CoO_x$ | 6.3 | This work |
| | 2.0 | Pt | 0.5 | – | 5 | [24] (2014) |
| $La_{0.5}Sr_{0.5}Ta_{0.5}Ti_{0.5}O_2N$ | 2.19 | 0.3 wt% Pt | 7.2 | – | 105 | [23] (2015) |
| $La_{0.75}Na_{0.25}TaO_{1.5}N_{1.5}$ | 2.76 | 0.3 wt% Pt | 7.8 | – | 4.9 | [54] (2013) |
| $BaTaO_2N$ | 1.80 | – | – | 2 wt% $CoO_x$ | 10.1 | [55] (2020) |
| $Y_2Ta_2O_5N_2$ | 2.2 | 0.15 wt% Pt+ 0.25 wt% Ru | 83.3 | – | 47 | [56] (2004) |
| $SrTaO_2N$ | 2.1 | 3 wt% Pt | 1.5 | 2 wt% $CoO_x$ | 22.5 | [57] (2017) |
| $Sr_2TaO_3N$ | 1.97 | – | – | 2 wt% $CoO_x$ | 6.2 | [58] (2018) |
| $SrNa_{0.2}Ta_{0.8}O_{2.8}N_{0.2}$ | 2.16 | – | – | 1 wt% $Rh_2O_3$ | 25.2 | [59] (2017) |
| $Ta_3N_5$ | 2.1 | – | – | 1 wt% $CoO_x$ | 78 | [33] (2015) |
| | | 0.02 wt% $Rh/Cr_2O_3$ | 3.5 | 0.02 wt% $Rh/Cr_2O_3$ | 1.7 | [12] (2018) |
| Zr doped $Ta_3N_5$ | 2.05 | – | – | 1 wt% $CoO_x$ | 212 | [60] (2016) |
| TaON | 2.5 | – | – | – | 165 | [61] (2002) |
| | | – | 110 | – | – | [62] (2015) |
| $CaNbO_2N$ | 2.0 | – | – | 2 wt% $CoO_x$ | 42.6 | [63] (2018) |
| Mg doped $CaNbO_2N$ | 2.11 | – | – | 3 wt% $CoO_x$ | 126.8 | [63] (2018) |
| $SrMg_{0.2}Nb_{0.8}O_{2+y}N_{1-y}$ | 1.97 | – | – | 1 wt% $CoO_x$ | 65.19 | [64] (2018) |
| $Sr_4Nb_2O_{9-x}N_x$ | 2.41 | – | – | 3 wt% $Rh_2O_3$ | 23 | [65] (2016) |
| $BaNbO_2N$ | 1.7 | – | – | 2 wt% $CoO_x$ | 15.3 | [66] (2016) |
| $LaTiO_2N$ | 2.1 | – | – | – | 44 | [67] (2018) |
| $La_{0.3}Ca_{0.7}TiO_{2.7}N_{0.3}$ | 2.0 | – | – | 1 wt% $CoO_x$ | 67 | [68] (2017) |

* Initial photocatalytic $H_2$ and $O_2$ evolution for 100 mg sample powders under visible-light (300 W Xe lamp, $\lambda \geq 420$ nm).

**Table S4** Summary of the photoluminescence decay lifetime ($\tau_1$, $\tau_2$), their relative amplitude ($f_1$, $f_2$) and amplitude weighted average lifetime ($<\tau>$) for $LaTaON_2$, $La_{0.1}Ca_{0.9}TaO_{1+y}N_{2-y}$ and $CaTaO_2N$; data are extracted by fitting the time-resolved PL spectra in Figure 4b by di-exponential functions.

| Sample | Decay lifetime (ns) | | Relative amplitude (%) | | Average lifetime ($<\tau>$, ns)* |
|---|---|---|---|---|---|
| | $\tau_1$ | $\tau_2$ | $f_1$ | $f_2$ | |
| $LaTaON_2$ | 1.93 | 33.95 | 45.55 | 53.45 | 32.44 |
| $La_{0.1}Ca_{0.9}TaO_{1+y}N_{2-y}$ | 1.45 | 50.68 | 16.05 | 83.95 | 50.41 |
| $CaTaO_2N$ | 0.55 | 6.11 | 56.19 | 43.81 | 5.53 |

*The average lifetime was calculated using equation: $<\tau> = (f_1\tau_1^2+f_2\tau_2^2)/( f_1\tau_1+f_2\tau_2)$.

**Table S5** The moles of photon flux per hour gauged by a quantum meter (Apogee MP-300).

| $\lambda$ / nm | Flux / μmol·h$^{-1}$ |
|---|---|
| 600 ± 40 | 2895 |
| 550 ± 35 | 2447 |
| 500 ± 35 | 1925 |
| 450 ± 35 | 1255 |
| 420 ± 20 | 1147 |

[47] A. C. Larson, R. B. Von Dreele, Los Alamos National Laboratory Report No. LA-UR-86-748 **1994**.

[48] P. Van der Heide, *X-ray photoelectron Spectroscopy - An introduction to principles and practices*, John Wiley & Sons, Inc, Hoboken, New Jersey **2012**.

[49] X. Q. Sun, X. X. Xu, *Appl. Catal. B-Environ.* **2017**, 210, 149.

[50] F. X. Zhang, A. Yamakata, K. Maeda, Y. Moriya, T. Takata, J. Kubota, K. Teshima, S. Oishi, K. Domen, *J. Am. Chem. Soc.* **2012**, 134, 8348.

[51] K. Maeda, K. Teramura, D. L. Lu, N. Saito, Y. Inoue, K. Domen, *J. Phys. Chem. C* **2007**, 111, 7554.

[52] S. S. Chen, S. Shen, G. J. Liu, Y. Qi, F. X. Zhang, C. Li, *Angew. Chem. Int. Edit.* **2015**, 54, 3047.

[53] G. V. Govindaraju, G. P. Wheeler, D. Lee, K. S. Choi, *Chem. Mater.* **2017**, 29, 355.

[54] K. Ueda, H. Kato, M. Kobayashi, M. Hara, M. Kakihana, *J. Mater. Chem. A* **2013**, 1, 3667.

[55] H. Zhang, S. H. Wei, X. X. Xu, *J. Catal.* **2020**, 383, 135.

[56] M. Y. Liu, W. S. You, Z. B. Lei, G. H. Zhou, J. J. Yang, G. P. Wu, G. J. Ma, G. Y. Luan, T. Takata, M. Hara, K. Domen, L. Can, *Chem. Commun.* **2004**, 2192.

[57] J. Fu, S. E. Skrabalak, *Angew. Chem., Int. Ed.* **2017**, 56, 14169.

[58] S. Wei, X. Xu, *Appl. Catal., B* **2018**, 228, 10.

[59] F. F. Wu, X. Q. Sun, G. Liu, X. X. Xu, *Catal. Sci. Technol.* **2017**, 7, 4640.

[60] Y. W. Wang, D. Z. Zhu, X. X. Xu, *ACS Appl. Mater. Interfaces* **2016**, 8, 35407.

[61] G. Hitoki, T. Takata, J. N. Kondo, M. Hara, H. Kobayashi, K. Domen, *Chem. Commun.* **2002**, 1698.

[62] Z. Wang, K. Xie, L. Zhao, B. S. Zhang, *Chem. Commun.* **2015**, 51, 2437.

[63] Y. W. Wang, S. Jin, X. Q. Sun, S. H. Wei, L. Chen, X. X. Xu, *Appl. Catal. B-Environ.*

**2019**, 245, 10.

[64] X. Q. Sun, G. Liu, X. X. Xu, *J. Mater. Chem. A* **2018**, 6, 10947.

[65] F. F. Wu, M. L. Lv, X. Q. Sun, Y. H. Xie, H. M. Chen, S. Ni, G. Liu, X. X. Xu, *ChemCatChem* **2016**, 8, 615.

[66] M. Hojamberdiev, E. Zahedi, E. Nurlaela, K. Kawashima, K. Yubuta, M. Nakayama, H. Wagata, T. Minegishi, K. Domen, K. Teshima, *J. Mater. Chem. A* **2016**, 4, 12807.

[67] Y. Zhang, J. Shi, C. Cheng, S. Zong, J. Geng, X. Guan, L. Guo, *Appl. Catal. B-Environ.* **2018**, 232, 268.

[68] F. Wu, G. Liu, X. Xu, *J. Catal.* **2017**, 346, 10.
